# Mapping nurses’ activities in surgical hospital wards: A time study

**DOI:** 10.1371/journal.pone.0191807

**Published:** 2018-04-24

**Authors:** W. F. J. M. van den Oetelaar, H. F. van Stel, W. van Rhenen, R. K. Stellato, W. Grolman

**Affiliations:** 1 Division of Internal Medicine, University Medical Center Utrecht, University of Utrecht, Utrecht, the Netherlands; 2 Julius Center for Health Sciences and Primary Care, University Medical Center Utrecht, University of Utrecht, Utrecht, the Netherlands; 3 Center for Human Resource Organization and Management Effectiveness, Business University Nyenrode, Breukelen, the Netherlands; 4 ArboUnie Occupational Health Service, Utrecht, the Netherlands; 5 Division of Surgical Specialties, University Medical Center Utrecht, University of Utrecht, Utrecht, the Netherlands; University Antwerp, BELGIUM

## Abstract

**Background:**

Balancing the number of nursing staff in relation to the number of patients is important for hospitals to remain efficient and optimizing the use of resources. One way to do this is to work with a workload management method. Many workload management methods use a time study to determine how nurses spend their time and to relate this to patient characteristics in order to predict nurse workload.

**Objective:**

In our study, we aim to determine how nurses spend their working day and we will attempt to explain differences between specialized surgical wards.

**Setting:**

The research took place in an academic hospital in the Netherlands. Six surgical wards were included, capacity 15 to 30 beds.

**Method:**

We have used a work sampling methodology where trained observers registered activities of nurses and patient details every ten minutes during the day shift for a time period of three weeks.

**Results:**

The work sampling showed that nurses spend between 40.1% and 55.8% of their time on direct patient care. In addition to this, nurses spend between 11.0% and 14.1% on collective patient care. In total, between 52.1% and 68% of time spent on tasks is directly patient related. We found significant differences between wards for 10 of the 21 activity groups. We also found that nurses spend on average 31% with the patient (bedside), which is lower than in another study (37%). However, we noticed a difference between departments. For regular surgical departments in our study this was on average 34% and for two departments that have additional responsibilities in training and education of nursing students, this was on average 25%.

**Conclusions:**

We found a relatively low percentage of time spent on direct plus indirect care, and a lower percentage of time spent with the patient. We suspect that this is due to the academic setting of the study; in our hospital, there are more tasks related to education than in hospitals in other study settings. We also found differences between the wards in our study, which are mostly explained by differences in the patient mix, nurse staffing (proportion of nursing students), type of surgery and region of the body where the surgery was performed. However, we could not explain all differences. We made a first attempt in identifying and explaining differences in nurses’ activities between wards, however this domain needs more research in order to better explain the differences.

## Introduction

Balancing the amount of nursing staff in relation to the amount of patients is important for hospitals to remain efficient [1]. Hospitals intend to deliver good quality of care and also work efficiently. To ensure this, there needs to be a good fit between patient needs and nursing staff on hospital wards. The amount of work that nurses do, their workload, needs to be well balanced, in order to prevent extra costs for overstaffing a ward but also to prevent deteriorating patient outcomes and increased stress or burnout in nurses by understaffing wards. There is a direct relation between nurses’ workload and patient outcome [2–5] and workload is also a predictor for burnout [6, 7]. Bakker found a relation between job demands such as workload and performance [8], and Toh’s study showed a positive bi-directional relation between the nursing shortage and oncology nurses' job dissatisfaction, stress and burnout [9]. Also, in the near future healthcare labor shortages are expected to occur [10], so retaining nursing staff will be a challenge. Workload is related to intention to leave [11, 12] and besides this, training of new staff is also costly. Many studies have identified factors that predict workload of nurses. There is evidence that these nurse-patient ratios or nursing hours per patient day (NHPPD) do not accurately predict workload of nurses [13], since these do not take into account the different needs between patients nor the differences in experience and education level of nursing staff. Twigg argues that relying on expert opinion in setting standards for workload, in their study a standard NHPPD per ward, is not optimal and recommends using a standardized patient acuity measurement [14].

In Belgium, hospitals are required to register the Belgium Nursing Minimum Data Set (B-NMDS) in order to benchmark hospitals on several dimensions, among which workload. Van den Heede showed that 70% of variation in nursing staff per unit was predicted by the B-NMDS item hospital type with the covariates nursing intensity and service type [15]. They recommended that instead of working with NHPPD, a NHPPD corrected for nursing intensity is a better measure. However, Sermeus stated in a 2008 study [16] that the B-NMDS nursing intensity did not necessarily give an indication of required nursing time. Another drawback of the B-NMDS is the extensive amount of registration required by the hospitals [17]. The RAFAELA™ patient classification system [18] is an instrument to assess optimum levels of nursing intensity. We consider this a form of workload management. The RAFAELA™ system consists of the Oulu Patient Classification instrument [19], a system that records daily nursing resources, and the Professional Assessment of Optimal Nursing Care Intensity Level questionnaire. The three are combined to measure nursing intensity. RAFAELA™ measures only the patient-related workload of nurses and does not include other tasks [20]. This method is widely used in Finland; while promising, it is not used for prospective workload management but only for assessments of workload in the past. For optimal versatility of nursing staff, prospective insight is of great value.

In a previous publication[21], we describe the development of a framework for a new workload management method. The first step in this method is to determine patient characteristics that are relevant to nurses’ workload. The second step in this method is to gain insight in what nurses’ activities are on a day to day basis. Quite some research has already been done in this area. In 2000, Rasmussen [22] selected examples of work sampling studies of nurses’ activities done in the 10 year time span from 1986 until 1996,. This overview showed results of studies in several settings (army hospital, regular hospital, different specialties including pediatrics and critical care), clustering activities in the categories Direct Care, Indirect Care (some studies have one category for the two), Unit-Related tasks and Personal Time (Prescott, [23]).

Duffield [24] performed a work sampling study of nurses and also worked with the same four different categories, as also used by Urden [25]. Direct care is defined as patient-related activities performed in the presence of the patient and indirect care is defined as patient-related activities away from the patient. It is assumed that patient-related activities can always be attributed to a single patient.

In 2008, Hendrich [26] performed a time- and motion study of nurses’ activities in 36 hospitals. Their goals were “to reveal drivers of inefficiency in how nurses spend their time and to identify opportunities to improve efficiency through changes to unit design and/or organization” [26]. With these goals in mind, nurses' time was divided into 4 categories of activities: nursing practice, unit-related functions, nonclinical activities, and waste. These 4 categories were in turn divided in a total of 12 subcategories. Unit-related functions were not divided in sub-categories of activities. However, unit-related functions also included patient related activities, such as transporting patients between wards. The subcategories were not specified, so subcategories of category Waste such as Looking/retrieving, Waiting and Delivering are difficult to interpret.

In 2011, Westbrook also performed a time and motion study [27], using the Work Observation Method by Activity Timing method. They focused on ten work tasks, amongst which direct care, indirect care and ward-related activities and social activities. These are partly the same as the 4 categories mentioned by Duffield, however some specific activities were classified under a separate work task, for example the engagement of nurses with other healthcare providers, supervision, documentation and medication activities. Activities within work tasks were not registered separately, the study registered activities on work task level.

In 1988, in the Netherlands, the Dutch Hospital Institute (NZi developed a workload management method using an activities list consisting of 23 activity groups which are clustered into categories Direct patient Care, Collective patient Care, Unit-Related tasks and Other time (which includes personal time and official breaks). These activity categories are quite similar to Prescott’s, but the clustering of activities under Direct patient care and Collective patient care is different. This was done with the purpose that all activities under Direct patient care can be linked to one specific patient. Activities under Collective patient care are often harder to attribute to one specific patient, for example collective preparation of medication or collective handover. When performing a time study with observation rounds done every ten minutes, each time an observation is done, 10 minutes of care time is attributed to the observed activity and also to the related patient. We believe that for some patient related activities like handover and collective preparation of medication, this would overestimate care time for certain patients and under estimate care time for other patients, because the time spent per patient is usually only a minute or two in these activities. In our study to develop a workload management method, we are interested in relating nurse care time to patient characteristics, so we are not primarily interested in where an activity took place or with whom, but if the activity and the related care time can be accurately related to a specific patient or not. We chose to use the NZi method as a starting point, because it fits this purpose. Also, the NZi list contains 23 activity groups, which is more than in other studies and helps us better evaluate and understand differences in working processes between nursing wards[28]. Lastly, several years ago, a small scale time study using the NZi method was performed in the same wards that are involved in the current study. Ward management and most nurses were still familiar with this list.

The current article describes a time study on activities of nurses, which is an important step in developing a new workload management method. We will describe how nurses spend their working day and the more detailed level of data collection will help understand differences between wards.

## Method

### Background

**Fig 1:**
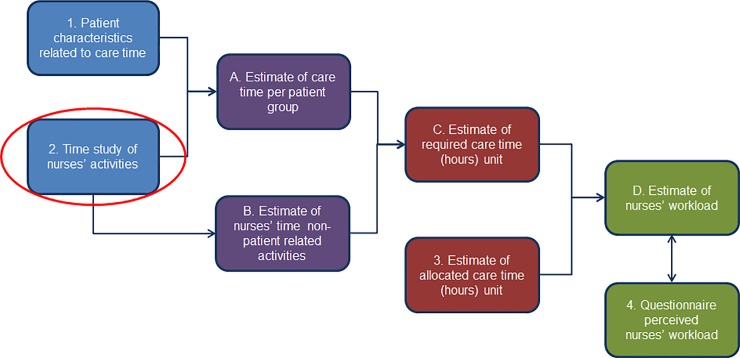
Developing a workload management method for staff nurses.

Performing a time study of nurses’ activities is the second of several steps in developing a workload management method for staff nurses. Fig 1 describes these steps. The full study protocol for developing this workload management method is described in our 2016 publication [21].

### Scope

The research took place in an academic hospital in the Netherlands. Six surgical wards were included, with 2 wards with 15 beds and 4 wards with 30 beds. Ward specialties were orthopedic/trauma surgery, vascular surgery, surgical oncology, otolaryngology, maxillofacial surgery and ophthalmology and urological surgery. The time study was done during the day shift. We chose to focus on the day shift, because this is the shift when the most nursing staff is required and most clinical nursing activities are performed. Weekends were excluded because task mix and staffing is very different in weekends and cannot be compared to day shifts of regular weekdays. We prioritized analyzing day shifts because controlling workload there will affect the most staff. In a later phase we plan to translate results to other shifts. Student nurses were included in the study; teamleaders and ward managers were excluded because they are not involved in direct nor indirect patient care.

Activities of other types of ward staff (doctors, assistants, cleaning staff, etcetera) were not considered in this study.

In our study, 87% of the nurses were female nurses, 62% were registered nurses and 38% nursing students. As shown in Table 1, 82% of the nurses is under 40 years of age.

**Table 1:** Age categories of nurses in study.

Type nurse/experience at study hospital (years)	<20	20–30	30–40	40–50	> = 50
**Registered Nurse**	29%	13%	9%	11%	0%
**Student Nurse**	37%	0%	0%	0%	0%

Of the registered nurses, 29% had less than 5 years’ work experience in the study hospital, 13% had 5–10 years’ experience and the others more than 10 years.

### Work sampling

To accurately map nurses’ activities, a work sampling methodology was used. Work sampling is a useful and efficient methodology to explore work-related activities [29]. In work sampling, activities of subjects are observed or registered every so many minutes, resulting in a sample of the activities of nurses. This way, we gain insight into the way nurses spend their working hours, for example to what extent their work is directly patient-related or which percentage of their working time is spent on administrative duties.

Pelletier and Duffield [29] suggest working with trained observers as an alternative to self-reporting, because the latter can be prone to bias. This is only possible when the staff to be observed works in an area that can be surveyed by the observer, and the observer can determine the activities relatively easily. For example, if work sampling is done on staff that is moving great distances or is performing mostly cognitive tasks, then self-reporting can be better. They also advocate the use of handheld computers to make registration faster and more accurate.

Sittig [30] also gives important tips for designing a work sampling study in healthcare: involve the nurses and nurse management in the study, determine relevant activities to register and make foolproof definitions, identify the right observers and train them well, and perform pilot samples to test the setup. We have followed up on these suggestions, in the next paragraphs we will elaborate on this.

#### Activities

Nurses perform many different activities in a day shift. Registering this multitude of activities separately is virtually impossible. We therefore first identified groups of activities that we wanted to register during the work sampling. The basis for this was the list of activity groups that is part of the workload management method developed by NZi (Dutch Hospital Institute, [31]). We used the Delphi method to evaluate the activity groups: the Delphi method is a structured form of communication in order to acquire an expert opinion on a certain topic [32]. In two or more rounds questionnaires are answered. In between rounds a facilitator gathers response and provides an anonymized summary of experts’ opinion, including the motivation of the experts. Experts can revise their opinion, based on the judgments of the other experts, working towards an end result with a good level of consensus.

Ward management selected one staff nurse of their ward to be the expert in the Delphi group. All selected nurses were experienced nurses in the specialty of the ward they work in.

The group was asked to comment on the NZi list of activity groups and corresponding activities. The activity groups were clustered into 4 categories: direct patient care, collective patient care, general tasks and other tasks. *Direct patient care* was defined as care time that can be directly related to one specific patient. This includes assistance with bathing or eating, handing out medication, changing bed linen, wound care, communication with the patient or family, etcetera. *Collective patient care* was defined as tasks that are patient-related, but are difficult to attribute to one specific patient. This includes general preparation of medication, patient handover, bringing a collection of samples to the laboratory, etcetera. *General tasks* includes education/supervision, meetings, organization of work (planning), administrative duties and domestic duties. *Other tasks* includes lunch and coffee breaks and personal time.

In one-on-one interviews with the lead researcher, members of the group commented on the list. Based on the group’s comments the list was adjusted: activities were added, group labels were adjusted and new groups were defined. Results were shared and the Delphi process was repeated, which resulted in a new, definitive activity group list (see Table 2). Note that this list shows only the activity groups and categories. Details of which activities are placed in the activity groups are not shown (data available on request).

**Table 2:** List of activity groups of nurses.

Activity Category	Activity group
**Direct patient care (DPC)**	1. Fluid/tissue sampling for laboratory research
** **	2. Assistance of doctors or others
** **	3. Communication with patient and/or family
** **	4. Oral communication/reporting about patient (not in presence of patient)
** **	5. Positioning and exercise
** **	6. Preparing/administering of medication (for individual patient)
** **	7. Observation of vital signs and routine check-ups
** **	8. Written/digital reporting, administration or transfer of information
** **	9. Transport of patient
** **	10. Personal care of patient
** **	11. Assistance meals and/or excretion
** **	12. Nurses' professional activities
**Collective patient care (CPC)**	13. Errands (away from ward)
** **	14. Collective handover of patient information (for multiple patients)
** **	15. Medication preparation (for multiple patients)
** **	16. Meals-related activities
**General tasks (GT)**	17. General administration
** **	18. Domestic activities
** **	19. Education and guidance
** **	20. Organization of work
** **	21. Meetings
**Other tasks (OT)**	22. Coffee break (authorized)
** **	23. Lunch break (authorized)
** **	24. Personal time

#### Observer selection and training

Observers were selected and trained uniformly in how to register nurses’ activities. Observers were either nurses from involved wards (observing on wards *other* than their own) or medical students. We preferred to work with nurses as observers where possible, because they are motivated to register activities accurately and are familiar with nurses’ activities, and therefore less likely to misinterpret or make mistakes. In addition, nurses learn about working procedures on other wards, which broadens their horizon and will help exchange ideas and increase understanding among wards. However, it was not possible to schedule sufficient nurses to cover all observer duties and we hired medical students where necessary. There were 49 observers in the study of which 18 were medical students.

Observer training consisted of a theoretical part (purpose of research, explanation of work sampling method, importance of accurate observation and registration) and a practical part (how to use the handheld computer, trial observations, examples of pitfalls). Attendance was mandatory for all observers and the training included a practical test under time pressure, and using all equipment that was to be used at the actual time study. Working instructions with all the work sampling study information were handed out to all observers. Observers were trained to always confirm with the nurses they were observing which activity the nurses were doing and -when relevant- for which patient. This procedure was introduced to prevent observers from making wrong assumptions.

#### Registration of observations

During the work sampling study all nurses were observed approximately every ten minutes. Observers registered their observations with a handheld computer (Symbol PDT-3100 barcode scanner, with SCO software), by scanning predefined barcodes. Time intervals between observations were automatically randomized, with an average of ten minutes. Observers were asked to register three things each time they made an observation: the name of the nurse; the activity the nurse was performing; and, when the activity was patient-related, patient details. There were three barcode sheets available to the observers. Each sheet showed all possible entries for one variable, in a logical order. One sheet showed barcodes for all names of the nurses, another showed all barcodes for the predefined activity groups and the last sheet showed barcodes for all patients on the ward. Barcodes were positioned in such a way on the sheet that chances of accidental miscoding were minimized. By registering which activities the nurses were doing every ten minutes, a random sample of nurses’ activities in day shifts was taken.

#### Test work sampling

Before doing the actual work sampling study, a test sampling study was performed. The purpose of this test study was to:

- Test the handheld computer and the barcode sheets: do they work properly and are they easy to use?
- Test the activities list: is it complete and easy to interpret?
- Test the workload of the observers: how many nurses can be observed by one observer and how long can an observer work uninterrupted?

We actively looked for flaws in the registration process so we could prevent registration errors in the actual work sampling study. Four observers received a uniform, standardized training and spent a minimum of three hours observing nurses on different wards. After this test study, the observers were interviewed. The equipment worked well and turned out to be reliable. Based on the observers’ experiences, choices were made regarding procedures:

- We changed the order and position of the barcodes to make them easier to locate and scan properly.
- A notepad was added to the observer’s equipment to note mistakes that could not be corrected on the spot.
- We decided to work with one observer per ward, with a maximum of 4 working hours in one observation shift.

#### Work sampling period

Next, the actual work sampling study was planned. A representative time period was selected, in which workload was expected to be average: outside holiday seasons and periods with especially high or low occupancy rates (for example, due to reduction of operating room capacity) and periods with enhanced or reduced nursing capacity (for example, due to planned education). Also, the number of observations that were to be made in the work sampling study needed to be sufficiently large. For practical reasons, there was a limit to the amount of days that we could observe our staff. It is costly to arrange observers, and nurses will get tired of being observed. Ward management was asked to advise on the maximum amount of time study days that they felt was reasonable, and they advised a maximum of fifteen consecutive working days. When sampling nurses’ activities every ten minutes, this would generate approximately 54,000 observations (= 15 study days x 6 wards x 12 nurses per ward x 50 observation rounds per day shift) of nurses’ activities. The actual number of observations of nurses’ activities was 54.663, which were aggregated to 290 observations of percentage of time spent on activities per nurse in a ward.

#### Work sampling study

All nurses on duty in the day shift of six wards were observed during the selected observation period of 15 working days (Monday through Friday). Trained observers registered activities approximately every 10 minutes in the day shift, starting at 07.30 hours and finishing at 16.00 hours. Exact observation moments and start and finish times were dependent on the random time interval generator of the handheld computer.

The standard training for observers included a procedure for correction of mistakes. If an observer made a mistake that could not be corrected on the spot, he or she would note details on the time, involved nurse and nature of the mistake on a note sheet on the clipboard. These notes were evaluated by the lead researcher, and checked by another researcher that was not directly involved in performing the work sampling. When corrections were approved by both researchers, they were corrected in the data. All corrections were logged uniformly.

#### Interrater agreement

To test whether two observers registered the same activity in the same way, an interrater agreement was determined. Regular tests for interrater agreement, such as Cohen’s Kappa or intraclass correlation cannot be applied here, because these assume that only one variable is observed and also that this variable is classified in a limited number of categories [33]. In our research, we have three variables (nurse/activity/patient), all with many possible categories: up to 15 names of nurses, 25 activities and up to 30 patients. For the study on differences of nurses’ activities between wards it is important that at least two variables (nurse name and activity group) were registered correctly. We decided to calculate an exact agreement percentage between the raters on these two variables.

To test the reliability of the registrations, an interrater agreement study was planned. The interrater agreement study was planned during the 3-week work sampling period. For this study, a second observer temporarily joined the scheduled observer. Both observers had the same training and both had already done at least one observation shift during the work sampling period. The agreement study was done twice, on two different wards with two different pairs of observers. One study was planned in the morning and one in the afternoon of the day shift. The observers walked their rounds in pairs and were instructed not to speak to each other or share registration results. On every observation round, one of the observers asked the nurses they observed which activity they were doing and, when applicable, for which patient. Both observers independently registered results in their own handheld computer. The interrater agreement was 88.4% exact agreement on 242 observations. We consider this an acceptable agreement percentage. The probability of an agreement occurring by chance is low, because there are so many selections possible for registration of nurse (15) and activity (25).

### Analysis

We analyzed our work sampling data in two steps:

#### 1. Descriptive analysis

This analysis gives a general impression of the way nurses spend their time, for the different wards that participated in the study. The descriptive analysis gives the mean percentage of time spent by nurses on the activity groups. However, this analysis does not give any information on variation within a department on the different activity groups, nor does it indicate whether observed differences could have been due to chance.

#### 2. Compositional analysis

We also studied whether there were statistically significant differences between wards on the time their nurses spent on the various activities. The times spent on different activities are correlated: if one increases, another must decrease, since the total always amounts to 100%. Compositional analysis is an appropriate method for such data, since it allows for correlated outcome variables that sum to a fixed total [34].

We first analyzed differences between wards on the activity categories: Direct Patient Care (DPC), Collective Patient Care (CPC), General Tasks (GT) and Other Tasks (OT). Compositional analysis dictates that one variable needs to be chosen as a reference variable, to compare the others against. The category OT was expected to be the most stable category, because the activity groups (duration of coffee and lunch breaks) that fall into this category are mostly standardized; therefore we chose OT as a reference category. The other three variables were compared to OT as follows: we calculated 3 ratios for each nurse in the study: DPC/OT, CPC/OT and GT/OT. Since ratios are difficult to handle mathematically and statistically [34], we converted the ratios to logratios. For each nurse in the study we defined three correlated logratios. The next 3 steps in the analysis were as follows:

1. **MANOVA on activity categories**. Since we had three correlated observations per subject (nurse), we used multivariate analysis of variance (MANOVA) to find significant differences on one or more of these variables between wards [34]. We used a significance threshold of 0.05 for the MANOVA.
2. **ANOVA on activity categories.** If the MANOVA indicated significant differences between wards, we wished to discover for which activity categories these differences materialize. This was done by an analysis of variance (ANOVA) for each of the three logratios separately. Again, a significance threshold of 0.05 was used.
3. **Post-hoc between wards on activity categories**. If the ANOVA indicated differences between wards on an activity category, then the next step was to make pairwise comparisons on each combination of wards for this activity category using a Tukey correction. This post-hoc test will indicate which wards differ from each other for time spent on a particular activity category.

After the analysis on activity categories, we performed a more detailed analysis in which we compared 21 separate activity groups to the reference category OT (the sum of three activity groups in category “Other Tasks”). Again, we first used a MANOVA on all activity groups and, if significant differences were found an ANOVA was performed separately for each activity group. Tukey post-hoc tests were carried out for activity categories for which the ANOVA indicated significant differences between the wards. Due to the large number of comparisons being made, we lowered the significance threshold to 0.01 for this analysis. The descriptive analysis was performed in Excel and the compositional analysis using the package “compositions” in R version 3.3.2 [35]. For help in interpretation, we discussed the results of the compositional analysis with the nurse managers.

### Ethical considerations

The study guaranteed the privacy of involved staff. There was no patient data recorded besides patient registration number. Only the lead researcher (lead author of this manuscript) has access to the master data and coded the data. Data have been processed in such a way that nothing can be traced back to specific persons. The study protocol was submitted to the medical ethical review board of the University Medical Center Utrecht and was approved, protocol number 14-165/C.

## Results

### Descriptive analysis

The mean percentage of time nurses spent on the 24 activity groups is shown in Table 3:

**Table 3:** Descriptive analysis of nurses' activities.

Activity Category	Activity group	Ward 1 Mean %	Ward 2 Mean %	Ward 3 Mean %	Ward 4 Mean %	Ward 5 Mean %	Ward 6 Mean %
**Direct patient care (DPC)**	1. Fluid/tissue sampling for laboratory research	0.8	0.9	0.5	0.4	0.3	0.3
** **	2. Assistance of doctors or others	2.1	1.8	2.2	1.4	1.4	1.4
** **	3. Communication with patient and/or family	7.6	4.4	4.2	4.2	5.1	7.8
** **	4. Oral communication/reporting about patient (not in presence of patient)	3.7	2.8	4.0	5.0	3.4	3.9
** **	5. Positioning and exercise	1.1	1.1	1.3	1.9	0.6	0.5
** **	6. Preparing/administering of medication (for individual patient)	6.6	4.3	3.6	3.2	2.9	1.9
** **	7. Observation of vital signs and routine check-ups	1.7	2.1	1.9	1.7	1.9	1.4
** **	8. Written/digital reporting, administration or transfer of information	13.8	11.4	12.8	13.0	12.3	11.4
** **	9. Transport of patient	5.3	3.1	3.1	2.9	3.6	3.4
** **	10. Personal care of patient	6.1	10.0	6.7	11.9	5.2	4.1
** **	11. Assistance meals and/or excretion	2.4	2.0	2.0	3.2	0.9	1.2
** **	12. Nurses' professional activities	4.5	3.3	4.3	4.4	2.4	3.0
**Total DPC**	** **	**55.8**	**47.3**	**46.8**	**53.2**	**40.1**	**40.4**
**Collective patient care (CPC)**	13. Errands (away from ward)	0.4	0.3	0.5	0.8	1.3	1.3
** **	14. Collective handover of patient information (for multiple patients)	11.1	10.9	9.0	9.1	10.1	12.3
** **	15. Medication preparation (for multiple patients)	0.7	1.8	1.4	2.1	0.6	0.4
** **	16. Meals-related activities	0.0	0.0	0.1	0.0	0.1	0.1
**Total CPC**	** **	**12.2**	**13.1**	**11.0**	**12.1**	**12.0**	**14.1**
**General tasks (GT)**	17. General administration	0.4	1.2	0.6	0.9	0.9	0.7
** **	18. Domestic activities	2.4	1.6	3.0	3.0	3.2	2.8
** **	19. Education and guidance	9.8	10.7	9.7	5.1	16.6	16.2
** **	20. Organization of work	1.3	3.7	3.8	1.7	2.2	5.9
** **	21. Meetings	2.7	2.5	6.6	3.7	7.5	4.1
**Total GT**	** **	**16.5**	**19.7**	**23.7**	**14.5**	**30.3**	**29.7**
**Other tasks (OT)**	22. Coffee break (authorized)	3.0	5.3	6.2	5.9	4.1	4.6
** **	23. Lunch break (authorized)	8.9	10.7	9.2	10.0	9.2	8.1
** **	24. Personal time	1.6	0.8	1.7	1.4	3.0	1.9
**Total OT**	** **	**13.5**	**16.8**	**17.1**	**17.2**	**16.3**	**14.6**
** **	25. Not found	2.0	3.1	1.4	3.0	1.3	1.2
**Total not found**	** **	**2.0**	**3.1**	**1.4**	**3.0**	**1.3**	**1.2**

Note: measurements in Table 3 were only for nurses that involved in direct care for patients. Team leaders and care assistants were excluded here, because not every ward has care assistants and on some wards team leaders spend much more time caring for patients than in other wards.

### Compositional analysis

We will show results for the activity categories and activity groups in separate paragraphs.

#### Compositional analysis activity categories

The MANOVA on the activity categories indicated significant differences between wards (p <0.001) and the ANOVAs detected significant differences between wards on all three categories. The post-hoc tests showed significant differences for many different combinations of wards, see Table 4.

**Table 4:** ANOVA activity categories.

	P-value (<0.05)		
Wards	DPC	CPC	GT
1–2	<0.001	0.251	0.784
1–3	<0.001	0.013	0.818
1–4	0.015	0.194	0.697
1–5	<0.001	0.186	0.128
1–6	0.002	0.977	0.020
2–3	0.997	0.773	0.058
2–4	0.949	0.999	0.999
2–5	0.975	0.999	<0.001
2–6	0.999	0.030	<0.001
3–4	0.788	0.867	0.038
3–5	0.999	0.944	0.706
3–6	0.999	<0.001	0.250
4–5	0.628	0.999	<0.001
4–6	0.939	0.021	<0.001
5–6	0.993	0.023	0.977

Ward 1 differed from all other wards on time spent on Direct Patient Care (DPC) in proportion to Other Time (OT). Since OT was relatively stable across wards, we can conclude that nurses on ward 1 spent significantly more time on DPC than nurses on other wards. The descriptive analysis suggested that ward 1 spent less time on General Tasks (GT) than the other wards, but only the difference between ward 1 and 6 was significant in the post-hoc comparison.

#### Compositional analysis activity groups

The MANOVA analysis on the activity groups resulted in a p-value of < 0.001, implying differences in time spent on activity groups between wards. The ANOVA per activity group detected significant differences between wards on many activity groups and the post-hoc results indicated which wards differed from one another. These results were added to the descriptive analysis and are displayed in **Table 5:**

**Table 5:** ANOVA activity groups.

Activity Category	Activity group	Ward 1 Mean %	Ward 2 Mean %	Ward 3 Mean %	Ward 4 Mean %	Ward 5 Mean %	Ward 6 Mean %	Differences between wards (p<0.01 posthoc)*
**Direct patient care (DPC)**	1. Fluid/tissue sampling for laboratory research	0.8	0.9	0.5	0.4	0.3	0.3	4–2
** **	2. Assistance of doctors or others	2.1	1.8	2.2	1.4	1.4	1.4	**No**
** **	3. Communication with patient and/or family	7.6	4.4	4.2	4.2	5.1	7.8	2–1, 3–1, 4–1, 5–1, 6–2, 6–3, 6–4
** **	4. Oral communication/reporting about patient (not in presence of patient)	3.7	2.8	4.0	5.0	3.4	3.9	**No**
** **	5. Positioning and exercise	1.1	1.1	1.3	1.9	0.6	0.5	**No**
** **	6. Preparing/administering of medication (for individual patient)	6.6	4.3	3.6	3.2	2.9	1.9	2–1, 3–1, 4–1, 5–1, 6–1, 6–2
** **	7. Observation of vital signs and routine check-ups	1.7	2.1	1.9	1.7	1.9	1.4	**No**
** **	8. Written/digital reporting, administration or transfer of information	13.8	11.4	12.8	13.0	12.3	11.4	**No**
** **	9. Transport of patient	5.3	3.1	3.1	2.9	3.6	3.4	3–1, 4–1
** **	10. Personal care of patient	6.1	10.0	6.7	11.9	5.2	4.1	6–2, 6–4, 5–4
** **	11. Assistance meals and/or excretion	2.4	2.0	2.0	3.2	0.9	1.2	5–1, 5–4
** **	12. Nurses' professional activities	4.5	3.3	4.3	4.4	2.4	3.0	5–1, 5–3
**Total DPC**	** **	**55.8**	**47.3**	**46.8**	**53.2**	**40.1**	**40.4**	** **
**Collective patient care (CPC)**	13. Errands (away from ward)	0.4	0.3	0.5	0.8	1.3	1.3	**No**
** **	14. Collective handover of patient information (for multiple patients)	11.1	10.9	9.0	9.1	10.1	12.3	**No**
** **	15. Medication preparation (for multiple patients)	0.7	1.8	1.4	2.1	0.6	0.4	6–2, 5–2
** **	16. Meals-related activities	0.0	0.0	0.1	0.0	0.1	0.1	**No**
**Total CPC**	** **	**12.2**	**13.1**	**11.0**	**12.1**	**12.0**	**14.1**	** **
**General tasks (GT)**	17. General administration	0.4	1.2	0.6	0.9	0.9	0.7	**No**
** **	18. Domestic activities	2.4	1.6	3.0	3.0	3.2	2.8	**No**
** **	19. Education and guidance	9.8	10.7	9.7	5.1	16.6	16.2	6–1, 6–2, 6–3, 6–4, 5–4
** **	20. Organization of work	1.3	3.7	3.8	1.7	2.2	5.9	**No**
** **	21. Meetings	2.7	2.5	6.6	3.7	7.5	4.1	5–1, 5–2, 5–4, 3–2
**Total GT**	** **	**16.5**	**19.7**	**23.7**	**14.5**	**30.3**	**29.7**	** **
**Other tasks (OT)**	22. Coffee break (authorized)	3.0	5.3	6.2	5.9	4.1	4.6	**= reference category**
** **	23. Lunch break (authorized)	8.9	10.7	9.2	10.0	9.2	8.1	**= reference category**
** **	24. Personal time	1.6	0.8	1.7	1.4	3.0	1.9	**= reference category**
**Total OT**	** **	**13.5**	**16.8**	**17.1**	**17.2**	**16.3**	**14.6**	** **
** **	25. Not found	2.0	3.1	1.4	3.0	1.3	1.2	
**Total not found**	** **	**2.0**	**3.1**	**1.4**	**3.0**	**1.3**	**1.2**	** **

*Note: the differences shown in the last column are the significant differences between wards on logratios of the activity group as compared to the reference category OT. Category “Not found” are missing data. Only significant differences are shown for the post-hoc tests.

**Direct patient care**:

- Fluid/tissue sampling: ward 2 (surgical oncology) spent more time on this than ward 4 (vascular surgery) due to frequent wound samples.
- Communication with patient/family: ward 6 (oral maxillofacial surgery) differs from all other wards, except for ward 5 (otolaryngology) and ward 1 (urology/ ophthalmology), and spends more time on communication than the others. This is likely because surgery in the maxillofacial area often leads to problems of speech. Ward 1 also spends more time on communication (differing from all but ward 6) due to the fact that many of the patients have vision problems. Nurses on this ward have to read labels and other information out loud to patients.
- Nurses’ professional activities: ward 5 (otolaryngology) spends less time on this than wards 1 (urology/ ophthalmology) and 3 (vascular surgery).
- Preparing medication: ward 1 (urology/ ophthalmology) spends more time on this than all other wards. Ward 6 (maxillofacial surgery) differs from ward 2 (surgical oncology) as well, it is unclear why.
- Transport of patient: ward 1 (urology/ ophthalmology) spends more time on this than wards 3 (vascular surgery) and 4 (traumatology/orthopedics), which can be explained by the complexity of the patient population and the resulting length of stay. Urology and ophthalmology patients typically have a short length of stay, and therefore more patients are admitted and transported to the operating rooms.
- Personal care of patient: ward 4 spends more time on this than wards 5 and 6, and ward 6 also spends less time on this task than ward 2. We could not explain these differences.
- Assistance with meals and/or excretion: ward 5 (otolaryngology) spends less time on this task than wards 1 (urology/ ophthalmology) and 4 (orthopedics, traumatology). Ear nose and throat surgery patients often cannot eat solid food (and therefore need no help in eating), whereas urology patients often need help with excretion by catheterization. The same goes for immobile patients from orthopedics and traumatology wards.

**Collective patient care**:

- Medication preparation: ward 2 (surgical oncology) spends more time on medication preparation than wards 5 (otolaryngology) and 6 (oral maxillofacial surgery).

**General tasks**:

- Education and guidance: ward 6 spends more time on this task than all other departments but ward 5, and ward 5 spends more time on this than ward 4. This can be explained by the fact that wards 5 and 6 together form a special learning environment, where a relatively high number of young nurses are trained.
- Meetings: ward 5 spends more time on this than wards 1,2 and 4 and ward 3 in turn spends more time on meetings than ward 2.

## Discussion

### Findings

The work sampling showed that nurses spent between 40.1% and 55.8% of their time on direct patient care. In addition to this, nurses spent between 11.0% and 14.1% on collective patient care. In total, this is between 52.1% and 68% of time spent on tasks that are directly patient-related.

We found significant differences between wards for 10 of the 21 activity groups. The biggest differences can be found for activity groups “Education/guidance” and “Medication preparation”, followed by activity groups “Assistance meals/excretion”, “Communication patient/family”, “Personal care” and “Meetings”. The results of the compositional analysis were used in discussions with ward managers on observed discrepancies between wards The diversity is mostly explained by differences in the patient mix, nurse staffing (proportion of nursing students), type of surgery and region of the body where the surgery was performed.

### Comparison to other work sampling studies

The NZi workload management method that we based our study on, employs a list of activity groups very similar to Duffield’s [24]. There is an important difference, though: NZi distinguishes a category called “Collective patient care (CPC)” which includes activities that are patient-related but cannot easily be attributed to a single patient. For example NZi classifies “Handover” as CPC because during a handover, each patient is discussed only for a short time. If during a work sampling study a handover meeting was observed and the full 10 minutes attributed to the patient being discussed at that moment, it would be an unfair allocation of time to that patient. In our study we were not only interested in the way nurses spend their time, but also in the relationship between nurses’ activities and patient characteristics (care time per patient group). Because of this, we chose to use a different list of activity groups and categories. We did not distinguish direct and indirect care activity groups on the basis of the location of the nurse (with the patient or away from the patient), but based on whether activities could be related to a single patient or not. Therefore we cannot directly compare our categories “Direct patient care” and “Collective patient care” to the categories “Direct care” and “Indirect care”. However, we can compare the sum of these categories.

In the studies shown in Rasmussen’s overview of work sampling studies of nurses’ activities [22], the sum of direct care plus indirect care makes up 59.7% to 67.6% of the activities of nurses. In our study, we found between 52.1% and 68% of nurses’ time was spent in these two categories, with an average of 60.9%. Our study was performed in a setting of surgical wards in an academic hospital, which is quite different to the settings in the studies mentioned by Rasmussen: amongst others a military hospital, critical care wards, psychiatric wards and pediatric wards. We suspect that part of the difference can be explained by the educational tasks inherent in the academic setting; as shown in Table 1, a substantial part of our workforce are nursing students: 38%. These students require education by the registered nurses, which explains part of the difference. In our study, wards with the highest percentage of time spent on educational activities also spent the least time on direct patient care.

Unit related tasks for the wards take up between 16 and 30% of nurses’ time in studies in Rasmussen’s overview[22]. In our research we found that General Tasks (which includes the same activity groups as unit-related tasks) comprised between 14.5% and 30.3% of time of the nurses. Personal time seems to vary considerably in Rasmussen’s overview: between 4% and as much as 20.7% of nurses’ time. Our study indicated less variation: between 13.5% and 17.2%. Personal time is quite standardized in our hospital: all nurses have one coffee break in the morning and a one-hour lunch break in the afternoon.

In 2008, Hendrich [26] performed a time- and motion study of nurses’ activities in 36 hospitals. We cannot compare our activity categories to this study, because the activity categories were too different from ours. For example, Hendrich defined a “Waste” category, which includes waiting, looking/retrieving and delivering. In our study, these activities were always related to a specific activity group. However, they concluded that nurses spend a smaller part of their time on patient care activities and more time on documentation, coordination of care, medication administration, and movement around the unit. This generally corresponds with our findings.

Westbrook also performed a time and motion study [27] in which observers shadowed nurses for blocks of, on average, one hour at a time. Westbrook found that the percentage of time spent on direct and indirect care (according to our definitions) was 76% and 81% in two consecutive measurements, which is much higher than in earlier studies. They also found that nurses spent around 37% of their time with patients. In our study, we found this to be on average 31%. However, we noticed a difference between departments. For regular surgical departments this was on average 34% and for the two departments that have additional responsibilities in training and education of nursing students, this was on average 25%. This explains why our average is much lower: the educational responsibilities in our academic setting influences how nurses spend their working day. This is interesting because there is evidence that the more time nurses spend with the patient, the higher the patient satisfaction [36] and the better outcomes [37–39].

We did not find any other study that analyzed the differences in time spent by nurses between different wards with different specialties. In our study we made a first attempt in identifying and explaining differences in nurses’ activities between wards, however we acknowledge this domain needs more research in order to better explain the differences and that these differences may vary between settings and countries.

### Study limitations

Our study was set in an academic hospital, which potentially limits the generalizability of the study results to different settings, such as general hospitals. Nurses’ activities in general hospitals may be different from activities in academic hospitals.

Also, the study was set in surgical wards; when applying the results to other specialties, adjustments will need to be made. Nurses on internal medicine wards spend their time on different activities than nurses on surgical wards. For example, wound care is not expected to be a predominant activity on internal medicine wards, but nurses there are likely to spend more time on blood transfusions, dialysis or chemotherapy, for example. Different specialties have different working processes, so our study results can be applied most easily to surgical wards. However, we expect that the method we used can be applied in any hospital, though it would likely result in different activity groups and different work sampling results.

One of our goals was to compare the percentage of time spent on activities between wards. For this purpose, we have 290 observations (observations of nurse/ward combination). This number allows for sufficiently detailed analysis of activities and differences between wards. The compositional analysis found significant differences on all levels, which supports this view.

Interrater agreement was 88.4%. There is no clear rule of thumb in literature that defines whether this is acceptable or not. Though our measure did not correct for accidental agreement, the chances of accidental agreement are very small due to the large number of categories for all three variables registered (15–25 per variable). Therefore we believe that 88.4% interrater agreement is sufficient.

We expected OT to be the most stable category, but there was still some variation between wards on this category. We did not find an explanation. However, we still stand by our choice to use this as a reference category, because it was the smallest category and the least interesting to compare across wards.

### Further research

More in-depth analysis is needed to study differences between wards that could not be readily explained.

As said, this work sampling study will also be used for developing a workload management system. The care time that nurses spent on specific patients will be related to patient characteristics that are expected to increase care time, such as isolation, psycho-social care or assistance with bathing. This way, we can calculate how much additional care time is needed when one or more of these characteristics applies to patients on a ward, forming the basis for a workload management method.

### Clinical implications and conclusion

The data collected in the work sampling study are very interesting from an operational excellence perspective. This study formed a basis for discussing the working processes of different wards and helped to identify and understand differences in processes and operational excellence between wards. The results can be analyzed further and provide a starting point for improvements. Results of this work sampling study will be combined with data on patient characteristics and lead to insight in required resources per patient and per ward. This, in turn, will be used to further develop a workload management method, as described in section 2.1.

## Supporting information

S1 Table Supporting information time study. (XLSX)

## Abbreviations

Nzi: Nationaal Ziekenhuis Instituut (Dutch Hospital Institute
DPC: Direct Patient Care
CPC: Collective Patient care
GT: General Tasks
OT: Other tasks
ANOVA: analysis of variance
MANOVA: multivariate analysis of variance
